# Evaluation of a Web Platform to Record Lifestyle Habits in Subjects at Risk of Developing Type 2 Diabetes in a Middle-Income Population: Prospective Interventional Study

**DOI:** 10.2196/25105

**Published:** 2022-01-17

**Authors:** Magdalena Del Rocio Sevilla-Gonzalez, Brigette Bourguet-Ramirez, Laura Sofia Lazaro-Carrera, Alexandro J Martagon-Rosado, Donaji Veronica Gomez-Velasco, Tannia Leticia Viveros-Ruiz

**Affiliations:** 1 Clinical and Translational Epidemiology Unit Massachusetts General Hospital Boston, MA United States; 2 Harvard Medical School Harvard University Boston, MA United States; 3 Unidad de Investigacion de Enfermedades Metabolicas Instituto Nacional de Ciencias Medicas y Nutrición Salvador Zubirán Mexico City Mexico; 4 Escuela de Medicina y Ciencias de la Salud Instituto Tecnológico y de Estudios Superiores de Monterrey Tec Salud Mexico City Mexico

**Keywords:** mHealth, prediabetes, type 2 diabetes, preventive medicine, diabetes, lifestyle, body mass index

## Abstract

**Background:**

Lifestyle is the focus of type 2 diabetes (T2D) prevention strategies. Prevention strategies using mobile health (mHealth)–based therapy have shown positive results for T2D prevention in high-income settings, but little is known about their effectiveness in low- and middle-income populations where the burden of T2D is substantial. “Vida Sana” is a web platform designed to record lifestyle habits and medication use within a lifestyle change program.

**Objective:**

We sought to identify the barriers, feasibility, usability, and effectiveness of Vida Sana to record lifestyle habits in subjects at risk of developing T2D in a middle-income setting.

**Methods:**

This was a 3-month prospective interventional study in Mexican individuals. A total of 77 subjects at risk of T2D (with prediabetes and BMI between 24 and 40 kg/m^2^) were selected. Feasibility was assessed by study retention. Usability was evaluated with the System Usability Scale (SUS). Effectiveness measures included changes in weight, body composition, BMI, glycated hemoglobin A_1c_ (HbA_1c_), and fasting blood glucose from baseline to 3 months. Linear regression models were used to account for covariates.

**Results:**

The feasibility of Vida Sana was 42%, with 33 subjects using the platform, and the usability was 48.7 (SD 14.24). Reported barriers to platform usage were; difficulty in accessing the platform from difficulty of use (12 subjects, 36%), lack of time to record their habits (11 subjects, 34%), lack of interest to record their habits (6 subjects, 18%), and lack of resources (4 subjects, 11%). The platform was effective for lowering glucose in fasting (–3.1 mg/dL vs –0.11 [SD 8.08] mg/dL; *P*=.038) and at 2 hours (–16.9 mg/dL vs 2.5 [SD 26.1] mg/dL; *P*=.045), body fat percentage (–1.3 [–2.2 to –0.7] vs –1.02 [–1.9 to –0.3]; *P*=.02), and waist circumference (–3.2 [SD 5.1] cm vs –1.7 [SD 5.0] cm; *P*=.02) independent of their age, sex, treatment, and education level.

**Conclusions:**

The use of the web platform was effective for improving glycemic and anthropometric parameters in a population at risk of developing diabetes. Improving accessibility and ease of navigation could improve the acceptance of digital health solutions in a middle-income population.

## Introduction

Type 2 diabetes (T2D) is a global health threat, and it is rapidly increasing worldwide, particularly in low- and middle-income countries [1]. T2D prevention has been declared a target priority by the World Health Organization [2] and the United Nations [3]. Target populations for prevention comprise people at high risk, such as those in the prediabetes state. Prediabetes is characterized by increased glucose concentrations without reaching T2D levels [4].

The most effective therapy to decrease T2D risk is lifestyle modification [5] and, in some cases, addition of pharmaceutical therapy [6]. Weight loss and improvements in body composition have been established as therapeutic targets in prediabetes management [6]. However, low adherence rates to lifestyle changes are major concerns in lifestyle modification trials [5,7-11]. Self-monitoring of lifestyle habits (ie, recording dietary intake, eating habits, physical activity, emotional state, sleeping hygiene, alcohol consumption, and smoking habits) is an effective tool to increase patients’ adherence to and awareness about their habits [6]. However, in some cases, the lack of a repository to track the records results in implementation difficulties.

Telemedicine has the potential to overcome barriers to treatment, such as self-monitoring, long distances to clinics, and long waiting times. Telemedicine is likely to continue growing and become more prevalent in medical care, especially due to the pressures placed on the health care system by the COVID-19 pandemic [12]. Most web-based interventions are more affordable than face-to-face therapy, allowing for broad dissemination of treatment, flexibility, and greater access. Although the use of health apps in dietary practice is common, the current digital applications for dietary counseling have not been extensively investigated [13]. The reports available describe high-income settings, and the effectiveness in other populations with different rates of metabolic disorders and social determinants of health is unknown. Hence, evidence regarding the barriers, feasibility, and effectiveness of these technologies is needed, particularly where the burden of T2D is substantial and access to highly trained health care professionals is limited. The aim of this study is to identify the barriers, feasibility, usability, and effectiveness of a web platform to record lifestyle habits in subjects at risk of developing T2D in a middle-income setting.

## Methods

### Study Participants

**Figure 1:**
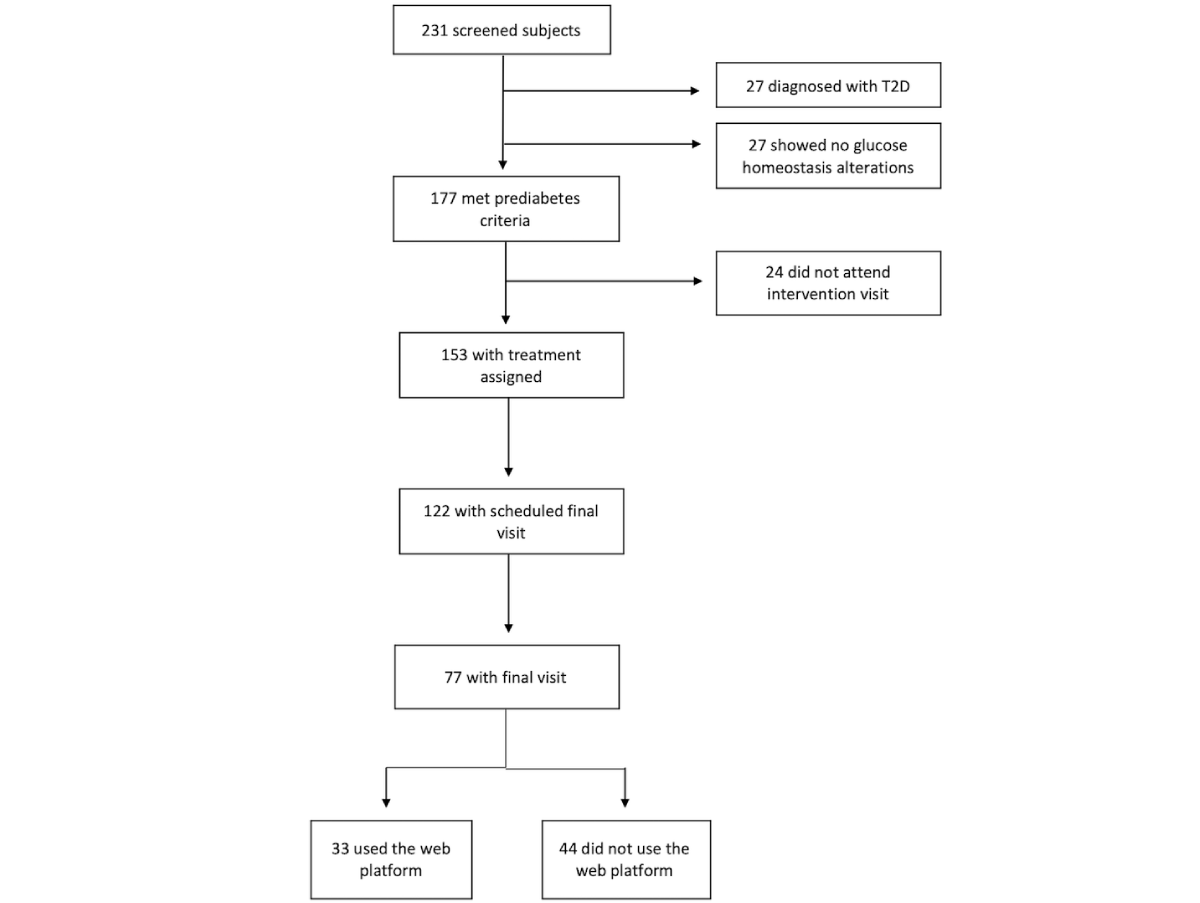
Number of individuals involved in the recruitment and screening process for selecting study participants. T2D: type 2 diabetes.

Participants were recruited at the Research Unit for Metabolic Diseases (UIEM) of the public hospital Instituto Nacional de Ciencias Médicas y Nutrición Salvador Zubirán (INCMNSZ) in Mexico City from September 2018 to March 2020, as outlined in Figure 1. Participants were invited through telephone calls as well as physical and electronic advertisements posted on the campuses and Facebook pages of UIEM and INCMNSZ, respectively. The prospective participants were subjects seeking treatment at the diabetes, obesity, internal medicine, or dyslipidemia outpatient clinics at the INCMNSZ, one of the most important institutions treating metabolic disorders in the country covering the largest population in the south of the Valley of Mexico. The criteria for selecting the study subjects were as follows: meeting at least 1 of the prediabetes criteria according to the American Diabetes Association, namely fasting glucose between 100 and 124 mg/dL, glycated hemoglobin A_1c_ (HbA_1c_) between 5.7 and 6.4, and 2-hour blood sugar level of 140 to 199 mg/dL after an oral load of 75 grams of glucose [4]. We included males and females aged between 18 and 65 years, and they were overweight or obese (BMI between 25 and 40 kg/m^2^).

Exclusion criteria included chronic diseases, pregnancy, chronic use of medications that alter plasma glucose levels, and subjects under or unable to maintain nutritional or physical activity therapies. The research protocol was approved by the ethics committee of the INCMNSZ. Written informed consent was obtained from each participant. Research was conducted according to the principles expressed in the Helsinki Declaration of Human Studies.

### Description of the Vida Sana Platform

**Figure 2:**
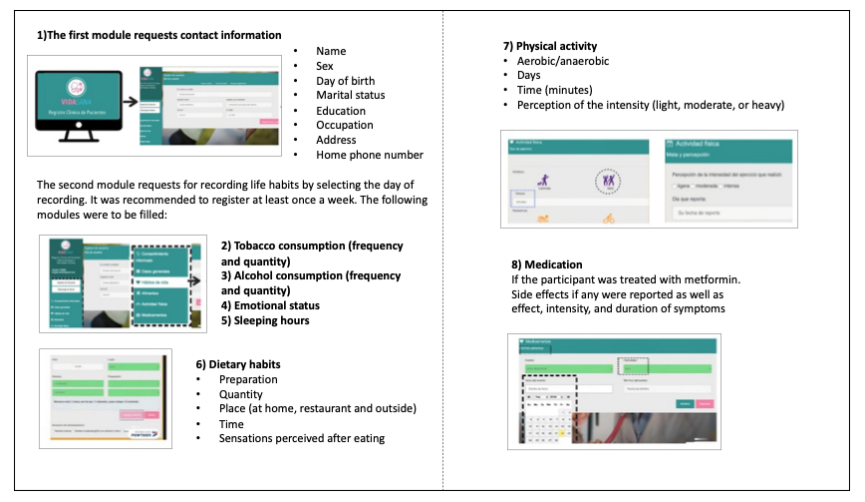
Vida Sana description. It is composed of 8 modules, 1 for personal information, and 7 for recording habits: tobacco consumption, alcohol consumption, emotional status, sleeping hours, dietary habits, physical activity, and medications.

Vida Sana is a web platform designed for this study by researchers of the Network for Research Support of the Universidad Nacional Autonoma de Mexico in 2017 to record lifestyle habits. User profiles were created for all participants using their email addresses. They accessed the platform either on a mobile phone or using a computer with internet. The dietitian explained the aims and scopes of the platform and trained the participants by filling an example record along with them during the first visit. In addition, participants were provided a video with instructions for using the web platform. The platform was composed of eight modules: (1) The first section in the first module asks for contact information, and the second section requests for recording lifestyle habits by selecting the day of recording. The other modules are as follows: (2) “tobacco consumption” indicating yes or no, and the number of cigarettes consumed if the answer is yes; (3) “alcohol consumption,” indicating the type of drink (options: beer, wine, liquor, and distilled) and the number of drinks; (4) predominant “emotional state” during the day (sad, anxious, angry, bored, guilty, happy, tired, and a blank space to type any other mood); (5) “hours of sleep,” (options: less than 4 hours, 4 hours, 5 hours, 6 hours, 7 hours, 8 hours, 9 hours, 10 hours, or more than 12 hours; and if they felt rested when awake). (6) “dietary habits” for recording the food consumed in 24 hours, splitting it into meals (breakfast, lunch, dinner, and snacks), and the time and place where the meals were consumed (house, restaurant, and work). Furthermore, the subjects are also required to enter the amount consumed in units (grams, pieces, cups, spoons, and milliliters) and the type of preparation (fried, weathered, breaded, roasted, boiled, raw, and stewed). Vida Sana also offers the option to select perceived sensations after eating (intense hunger, moderate hunger, neither hungry nor full, satisfied, and hard to digest). (7) “physical activity” for entering the type of exercise performed (options: aerobic exercises, such as walking, dancing, and aerobics; resistance exercises, such as swimming, cycling, garters, and weights; and flexibility exercises). Participants also had to enter the perception of the intensity with which they performed the exercise, namely light, moderate, or intense. (8) “medication” for entering the dose, frequency, and duration of intake, and secondary symptoms such as nausea, vomiting, abdominal pain, dizziness, diarrhea, constipation, and dry mouth, with the option of tracking the beginning of the symptoms (Figure 2).

### Study Procedures

This was a 3-month follow-up intervention trial, with 2 arms of intervention including lifestyle changes and lifestyle changes + metformin. The study covered six visits: screening, 4 intermediate visits, and 1 final visit. In the first visit, subjects underwent a 3-hour oral glucose tolerance test [14] and body composition assessment involving dual-energy X-ray absorptiometry to confirm compliance with the inclusion criteria. In addition, anthropometric measurements were recorded; waist and hip circumferences (to the nearest 0.5 cm) were measured at the midpoint between the lower ribs and the iliac crest, and at the level of the trochanter major, respectively. Participants who met the inclusion criteria were invited to return for the intervention visit in the following week.

The intervention included lifestyle modification counseling with the goal of reaching a weight loss of >3%. Nutritional and physical activity modifications were prescribed by a dietitian. The nutritional care process [15] was used to establish patients’ evaluations, diagnosis, and interventions. The strategies aimed to reduce caloric intake, with the following macronutrient distribution: 45% carbohydrates, 25% protein, and 30% lipids in the total caloric intake. The number of calories were determined by reducing 500 kcal from the total energy expenditure. The minimum and maximum calories established in the study were 1300 and 1900 kcal, respectively. Some individuals were randomly prescribed 750-mg extended-release metformin every 12 hours to evaluate the “medication” module of the web platform. During the intervention visit, patients were asked for their email accounts to give them access to the Vida Sana web platform.

Participants were asked to return every 2 weeks for follow-up visits to undergo lifestyle modification counseling for reinforcing their knowledge toward fulfillment of their treatment goals. These visits took place at the UIEM, according to the subjects’ availability of time. Body composition measurements were conducted using a bioimpedance instrument (SECA mBCA514).

The final visit took place 12 weeks after the intervention visit. Laboratory tests and body composition measurements were conducted, and questionnaires for diet and physical activity were used.

### Questionnaires and Calculations

Daily energy, and macronutrient and micronutrient intakes were assessed through a 24-hour food recall. Data were analyzed using ESHA’s Food Processor® Nutrition Analysis software. For baseline and final visits, body composition was assessed through dual- energy X-ray absorptiometry (General Electric); patients were asked to fast for a minimum of 4 hours. For assessing insulin resistance, we used the homeostasis model assessment for insulin resistance given by the following formula: glucose × fasting insulin/405 and the oral glucose insulin sensitivity index (OGIS) [16,17].

### Outcome Measurements

The feasibility of Vida Sana was assessed by self-reports through a single question asked to each of the participants: “Did you use the web platform during the study?” Barriers were evaluated by asking the following question to those who did not use the platform: “Which barriers did you find for not using Vida Sana?” Vida Sana’s usability was assessed with the System Usability Scale (SUS) [18] among individuals who reported usage of the platform at least twice during the study. Effectiveness measurements included changes in fasting glucose, glucose at 120 minutes, body fat percentage, waist circumference, visceral adipose tissue, and free-fat mass index. The intervention was not expected to pose any serious risk of adverse events (AEs) for participants. Participants were instructed to call the research team over telephone in case of an adverse event (AE) so that they could provide details for determining whether the event was related or unrelated to the intervention.

### Biochemical and Sample Analyses

Measurements derived from the oral glucose tolerance test included glucose levels, insulin, and lipid profiles, determined using colorimetric enzymatic methods (Unicel DxC 600 Synchron Clinical System, Beckman Coulter). Insulin was measured with a chemiluminescence essay (Access 2, Beckman Coulter). As for HbA_1c_, a 4-mL peripheral blood sample was drawn via venipuncture using the standardized technique and measured using a Variant II Turbo system (BIORAD); the method used was high pressure liquid chromatography.

### Statistical Analysis

Participants who did not attend their follow-up visits in the first 2 months and those with more than 20% of missing data were not considered during the analysis. The baseline characteristics of the study population were analyzed with descriptive statistics. Quantitative variables were reported as means and SDs for parametric variables, and medians and IQRs for nonparametric variables. Normality was assessed using the Kolmogorov-Smirnov test. Qualitative variables were presented as frequencies and percentages. For continuous outcome measures, the delta from baseline was calculated by subtracting the value of the baseline visit from that of the final visit. To estimate statistical differences between those who used Vida Sana and those who did not use it, a *t* test or Mann-Whitney *U* test was conducted according to the variable distribution. Differences before and after treatment were computed with a paired *t* test or Wilcoxon test according to the distribution. Fold changes were computed to account for baseline values. Lineal regression models were used to adjust for covariates using fold changes as the outcomes and age, sex, treatment, and education attainment as the confounders. All analyses were performed using the R software package (version 3.6.1; The R Project for Statistical Computing).

## Results

### Population Characteristics

The population baseline characteristics are given in Table 1. A total of 231 subjects were screened to participate in the study; however, the recruitment was interrupted by the COVID-19 pandemic, and only 77 individuals completed the 3-month intervention. The population was mainly composed of women (54/77, 70%). The mean age of the final sample was 48.45 (SD 11.32) years, and the most frequently altered diagnostic criterion was HbA_1c_ in 33 subjects (42%) with a mean of 5.90% (SD 0.26%). The lifestyle modification arm comprised 45 subjects (58%), whereas the metformin + lifestyle modification arm comprised 33 subjects (43%). Eating habits of the participants reflect unhealthy habits such as deficiency in fiber intake with a mean consumption of 16.75 (SD 16.51) grams, indicating a lower fiber consumption compared with that given in the World Health Organization guidelines [19]. Likewise, excess consumption of simple sugars was observed, with a median of 49.89 grams, exceeding the recommended daily intake of 25 grams [19].

**Table 1:** Baseline population characteristics (N=77)^a^.

Characteristic		Value
Age (years), mean (SD)		48.4 (11.3)
Male, n (%)		24 (30)
**Education attainment, n (%)**		
	Elementary school diploma	4 (5.1)
	Junior high school diploma	7 (9)
	High school diploma	17 (22)
	Technician degree	3 (3.8)
	Bachelor’s degree	37 (48)
	Graduate school degree	9 (11.6)
**Treatment, n (%)**		
	Lifestyle modification	45 (57.6)
	Lifestyle modification + metformin	33 (42.3)
Fasting glucose (mg/dL), mean (SD)		97 (9.6)
Glucose after 120 minutes (mg/dL), mean (SD)		127 (26.2)
HbA_1c_^b^ (%), mean (SD)		5.9 (0.2)
Fasting insulin (U/mL), mean (SD)		7.9 (4.2)
Cholesterol (mg/dL), mean (SD)		188.7 (37.6)
Triglycerides (mg/dL), mean (SD)		151 (92)
HOMA^c^, mean (SD)		1.9 (1.1)
OGIS^d^, mean (SD)		404.4 (77.0)
Weight (kg), mean (SD)		78.8 (13.4)
BMI (kg/m^2^), mean (SD)		30.4 (4.4)
Waist circumference (cm), mean (SD)		98.5 (11.4)
WHR^e^, mean (SD)		0.9 (0.07)
Body fat (%), mean (SD)		40.2 (7.0)
Free-fat mass index, mean (SD)		17.0 (2.0)
VAT^f^ (g), mean (SD)		1219.5 (488.5)
Daily caloric intake (kcal), mean (SD)		1863.1 (1193.9)
Daily protein consumption (%), mean (SD)		17.6 (6.5)
Daily fat consumption (%), mean (SD)		31.1 (9.0)
Carbohydrate consumption (%), mean (SD)		51.4 (12.6)
Daily average sugar consumption (g), mean (SD)		49.8 (52.5)
Daily average fiber consumption (g), mean (SD)		16.7 (16.5)
Sleep (hours), mean (SD)		6.4 (1.1)

^a^Variables are presented as means and SDs or medians and IQRs according to the variable distribution.

^b^HbA_1c_: glycated hemoglobin A_1c_.

^c^HOMA: homeostasis model assessment.

^d^OGIS: oral glucose insulin sensitivity index.

^e^WHR: waist-hip ratio.

^f^VAT: visceral adipose tissue.

After the 3-month intervention, 4 of the 77 subjects (5.12%) progressed to T2D, whereas prediabetes regression was observed in 9 subjects (12%); 64 subjects (82%) remained in the prediabetes state. Overall, 44 subjects (56%) reached the total body weight loss goal of >3%. The average weight loss per person was 2.92 (SD 2.77) kg.

### Feasibility and Usability

Out of the 77 subjects, 33 (42%) reported using Vida Sana. Among the subjects who did not use the platform, the main barriers reported were classified into four main categories: difficulty in accessing the web platform owing to difficulties in using the platform (16, 36%), lack of time to record their habits (15, 34%), lack of interest to record their habits (8, 18%), and lack of resources (computer or internet) (5, 11.36%). The usability of Vida Sana was estimated to be 48% (SD 14.24%) (low usability) according to the SUS. Through the questionnaire, the subjects reported the following weaknesses and strengths: The better functioning of the web platform on a computer than on a mobile phone made the recording process complicated. Similarly, the number of sections to be completed was found to be considerably high. Lastly, having to type the answers instead of selecting options made the recording process cumbersome. The positive aspects were that the web platform did not need detailed usage training or previous experience using electronic applications.

Table 2 presents the participant details based on their usage of Vida Sana at baseline. Statistically significant differences were found in fasting glucose concentrations (*P*=.04) and education attainment (*P*=.006).

**Table 2:** Baseline characteristics of the population based on platform usage (N=77)^a^.

Characteristics			Used web platform (n=33)		Did not use web platform (n=44)		*P* value^b^	
Age (years), mean (SD)			48 (12.0)		48.4 (10.8)		.89	
Male, n (%)			12 (36.3)		12 (27.2)		.39	
**Education attainment, n (%)**								.006
	Elementary school diploma	0 (0)		4 (9)				
	Junior high school diploma	0 (0)		7 (15.9)				
	High school diploma	4 (12.1)		13 (29.5)				
	Technical degree	2 (6)		1 (2.2)				
	Bachelors’ degree	22 (66.6)		15 (34)				
	Graduate school degree	5 (15.1)		4 (4)				
**Treatment, n (%)**								.18
	Lifestyle modification	6 (48.4)		28 (63.6)				
	Lifestyle modification +metformin	17 (51.5)		16 (36.3)				
Fasting glucose (mg/dL), mean (SD)			94.6 (9.0)		99 (9.6)		.05	
Glucose at 2 hours (mg/dL), mean (SD)			125.2 (28.1)		130 (24.9)		.44	
HbA_1c_^c^ (%), mean (SD)			5.8 (0.3)		5.9 (0.2)		.52	
HOMA^d^, mean (SD)			1.7 (0.9)		2.0 (1.3)		.22	
OGIS^e^, mean (SD)			406 (86.1)		403 (70.1)		.87	
Weight (kg), mean (SD)			81.4 (16.5)		78.5 (13.7)		.40	
BMI (kg/m^2^), mean (SD)			30.8 (16.5)		30.6 (4.2)		.87	
Waist circumference (cm), mean (SD)			98.6 (12.3)		99.2 (11.8)		.84	
WHR^f^, mean (SD)			0.91 (0.7)		0.92 (0.0)		.68	
Body fat (%), mean (SD)			32.5 (7.1)		40.7 (6.9)		.44	
Free-fat mass index, mean (SD)			17.2 (2.2)		16.8 (1.8)		.41	
VAT^g^ (g), mean (SD)			1269 (609)		1183.6 (381)		.48	
Daily caloric intake (kcal), mean (SD)			1228 (1313)		2031 (1129)		.89	
Daily caloric protein intake (%), mean (SD)			18.2 (4.1)		16.3 (7.5)		.21	
Daily lipid intake (%), mean (SD)			31.2 (10.3)		31.4 (12)		.97	
Carbohydrate consumption (%), mean (SD)			51.7 (12.9)		50.9 (11.9)		.99	
Sugar consumption (g), mean (SD)			65.1 (42.3)		55.6 (40.8)		.32	
Fiber consumption (g), mean (SD)			16.7 (14.7)		17.5 (17.6)		.75	
Sleep (hours), mean (SD)			6.3 (0.9)		6.5 (1.1)		.39	

^a^Variables are presented as means and SDs or medians and IQRs according to their distribution.

^b^*P* values were calculated from Student *t* or Mann-Whitney *U* tests according to the distribution.

^c^HbA_1c_: glycated hemoglobin A_1c_.

^d^HOMA: homeostasis model assessment.

^e^OGIS: oral glucose insulin sensitivity index.

^f^WHR: waist-hip ratio.

^g^VAT: visceral adipose tissue.

### Effectiveness and Safety

**Figure 3:**
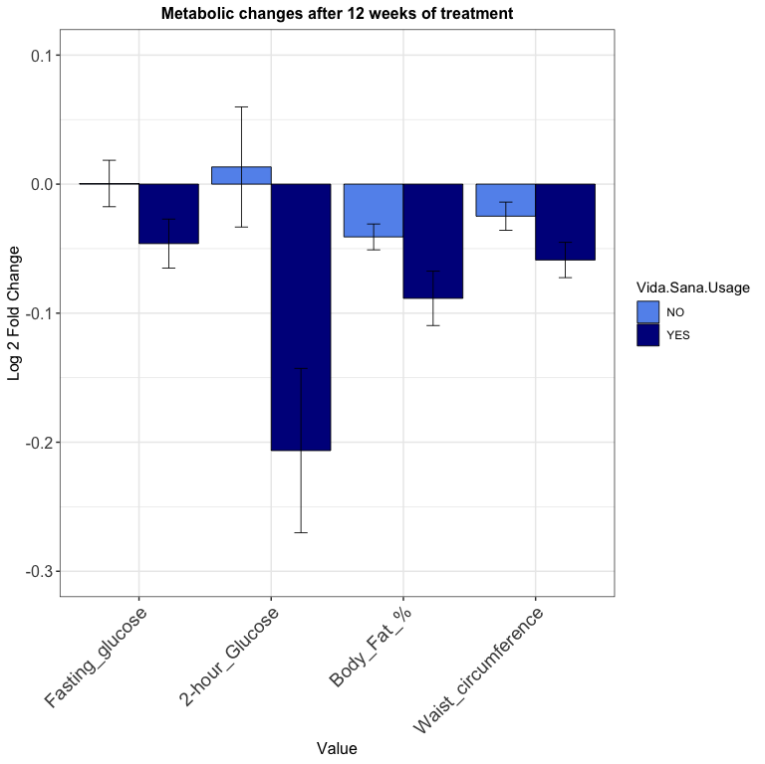
Changes in metabolic parameters after 12 weeks of treatment according to platform usage. Log 2 fold changes are presented. *P* values were computed with a linear regression model using fold changes as outcomes, and age, sex, treatment, and education attainment as covariates. Vida Sana implementation after 3 months of intervention showed a significant decrease in fasting glucose (*P*=.03), 2-hour glucose (*P*=.04), body fat percentage (*P*=.024), and waist circumference (*P*=.023).

Anthropometric, biochemical, and eating habit changes after the intervention are shown in Figure 3. Implementation of Vida Sana after 3 months of intervention showed a significant decrease in fasting glucose, 2-hour glucose, body fat percentage, and waist circumference after adjusting for age, sex, treatment, baseline values, and education attainment, as observed in Table 3.

Others showed interesting clinically relevant changes without statistical significance; there was a reduction in the daily carbohydrate and sugar intake among subjects who used Vida Sana. Conversely, there was an increase in daily protein intake and lean body mass in those who did not use the platform. However, these were not statistically significant (Table 3). There were no AEs reported in this study.

**Table 3:** Changes in metabolic parameters after 12 weeks of intervention by platform usage (N=77).

Change in metabolic parameter	Used the web platform (n=33)	Did not use the web platform (n=44)	*P* value^a^	Adjusted *P* value^b^
Δ^c^ Weight (kg)**,** mean (SD)	–3.4 (3.1)	–2.5 (2.3)	.13	.19
Δ BMI (kg/m^2^), mean (SD)	1.2 (1.1)	–0.9 (0.9)	.24	.28
Δ Body fat (%)	–1.3 (–2.2 to –0.7)	–1.0 (–1.9 to –0.3)	.12	.02
Δ Free-fat mass index, mean (SD)	–0.1 (0.5)	–0.09 (0.4)	.51	.47
Δ VAT^d^ (g), mean (SD)	–177 (232.3)	–35.5 (187.9)	.008	.12
Δ Waist circumference (cm), mean (SD)	–3.9 (5.1)	–1.7 (5.0)	.06	.02
Δ HbA_1c_^e^ (%), mean (SD)	–0.07 (0.2)	0.01 (0.3)	.26	.35
Δ Glucose at 0 minutes or baseline (mg/dL), mean (SD)	–3.1 (7.0)	–0.1 (8.0)	.08	.03
Δ Glucose at 120 minutes (mg/dL), mean (SD)	–16.9 (29.5)	2.5 (26.1)	.03	.045
Δ Daily caloric intake (kcal), mean (SD)	–116.4 (1136)	–427.3 (1001)	.71	.20
Δ Daily protein intake (%), mean (SD)	1.4 (7.2)	1.0 (7.8)	.68	.59
Δ Daily lipid intake (%), mean (SD)	1.1 (12.3)	–1 (9.2)	.82	.66
Δ Carbohydrate consumption (%), mean (SD)	–3.6 (15)	0.10 (14.8)	.49	.49
Δ Sugar (g), mean (SD)	–20.0 (48.7)	–7.8 (43.0)	.25	.69
Δ Fiber (g), mean (SD)	–0.08 (15.8)	–3.2 (15.7)	.96	.22
Δ Sleeping hours, mean (SD)	0 (0 to 1.0)	0 (0 to 1.0)	.52	.31

^a^*P* values were calculated from paired *t* or Mann-Whitney *U* tests according to the variable distribution.

^b^Adjusted *P* values were computed with a linear regression model using fold changes as outcomes, and age, sex, treatment, and education attainment as covariates.

^c^Δ: change.

^d^VAT: visceral adipose tissue.

^e^ HbA_1c_: glycated hemoglobin A_1c_.

## Discussion

Our study showed that Vida Sana was effective in lowering glucose in fasting, 2-hour glucose, body fat percentage, and waist circumference independent of age, sex, treatment, and education attainment. We found a feasibility of 42.8% (33 subjects) and a usability level of 48.71 (SD 14.24) for Vida Sana. To our knowledge, this is the first study conducted in Mexico and one of the first studies in Latin America to determine the barriers associated with and effectiveness of telemedicine as part of a nutritional intervention.

Our study provides important evidence on the barriers that arise when implementing mobile health (mHealth) technologies in a middle-income country. Although diabetes is the most commonly targeted medical condition for implementing mHealth technologies, implementation remains inadequate [20]. We found that the main barrier was the difficulty in accessing the web platform, as the subjects found it difficult to use. Previous studies [21-23] have indicated similar barriers such as the lack of infrastructure, lack of equipment, and technology gap while addressing security and privacy issues, illiteracy, technical problems, costs, and financial sustainability [24]. Conversely, the reported key factors associated with the success of telemedicine programs are integrating these programs with existing systems, minimizing the burden for health care providers, and applying user-friendly technology [25]. Considering the barrier of technology gap, our study proved that education level was an important determinant for platform usage. It is well known that lower rates of education attainment in low- and middle-income countries, which are critically correlated with the familiarity in using technologies. To overcome this barrier, we believe it is critical to invest in training to increase familiarity with the platform, particularly in subjects with low education attainment. This would also help overcome the second barrier, namely lack of interest to use the tool. Training people in using the tool coupled with an orientation on the importance of self-monitoring and self-efficacy are strategies that would improve the feasibility and acceptance of such platforms. The lack of resources such as time turned out to be a major barrier; therefore, it is important to consider scheduling the most appropriate times for data recording with the patients. Additionally, features that could help increase interest in recording habits include sending reminders, designing friendly interfaces, acknowledging the users’ achievements, and visually tracking their records.

In this study, we determined a feasibility rate of 42% (33 subjects), which is lower than that observed in studies with similar inclusion criteria and study designs but different populations, in which the feasibility rates reached 71.1% [26] and 86% [27]. The platform developed by Everett [27] had some important different features such as personalized automatic notifications through a machine learning algorithm according to the patients’ habits that could help to improve the app’s usability. In contrast, the platform described by Brock [26] used a website with the aim of providing behavioral support to improve physical activity, eating habits, and factors such as weight loss, stress, and sleep. Our platform differed slightly in this regard. We sent reminders during the week by email and via mobile phone to the participants that could help improve the feasibility and usability of the platform.

Despite its low usability, Vida Sana was effective in reducing metabolic parameters such as fasting glucose, 2-hour glucose, body fat percentage, and waist circumference, independently of age, sex, baseline values, treatment, and education attainment. This result is consistent with the findings from a previous systematic review indicating that 50% of the eHealth and mHealth interventions were effective in increasing physical activity, and 70% of the identified interventions were effective in improving diet quality [28]. However, despite the highly positive results shown by a systematic review of randomized controlled trials, reaching a clear conclusion about the effectiveness of mHealth interventions against noncommunicable diseases is not yet possible because of the limited number of studies, heterogeneity of the evaluated mHealth interventions, and wide variety of reported outcome measures [28].

The strengths of the study lie in the appropriate clinical characterization of the subjects with prediabetes; we evaluated the barriers, feasibility, usability, and effectiveness associated with an intervention in a single study. We developed a standardized, qualified nutritional intervention that helps reduce the risk of bias in terms of the quality and variability of the intervention, described as an important aspect of treatment effectiveness. Our sample is diverse with varying education levels and age groups although it predominantly comprised women. The follow-up time during the 3 months allowed us to evaluate adherence in the short term. These findings may be somewhat limited by the sample size. Therefore, further studies with more focus on improving usability, longer follow-ups, and diverse disease outcomes are suggested.

These results reinforce the effectiveness of telemedicine in nutritional and lifestyle interventions. It shows that even with low usability and feasibility, telemedicine is effective in improving glycemic and anthropometric parameters in a population at risk of diabetes. Improving accessibility, ensuring easy navigation, and providing an orientation regarding the potential benefits of using technology should be the objectives of future research to improve the acceptance of mobile apps in a middle-income setting.

## Abbreviations

AE: adverse event
HbA_1c_: glycated hemoglobin A_1c_
INCMNSZ: Instituto Nacional de Ciencias Médicas y Nutrición Salvador Zubirán
mHealth: mobile health
OGIS: oral glucose insulin sensitivity index
SUS: System Usability Scale
T2D: type 2 diabetes
